# Zebrafish as a Model for the Study of Human Myeloid Malignancies

**DOI:** 10.1155/2015/641475

**Published:** 2015-05-03

**Authors:** Jeng-Wei Lu, Meng-Shan Hsieh, Heng-An Liao, Yi-Ju Yang, Yi-Jung Ho, Liang-In Lin

**Affiliations:** ^1^Department of Clinical Laboratory Sciences and Medical Biotechnology, College of Medicine, National Taiwan University, No. 1 Chang-Te Street, Taipei 100, Taiwan; ^2^Institute of Preventive Medicine, National Defense Medical Center, No. 172 Dapu Road, New Taipei City 237, Taiwan; ^3^Graduate Institute of Life Sciences, National Defense Medical Center, No. 161, Section 6, Minquan East Road, Taipei 114, Taiwan; ^4^Department of Laboratory Medicine, National Taiwan University Hospital, No. 7 Chung-Shan Southern Road, Taipei 100, Taiwan

## Abstract

Myeloid malignancies are heterogeneous disorders characterized by uncontrolled proliferation or/and blockage of differentiation of myeloid progenitor cells. Although a substantial number of gene alterations have been identified, the mechanism by which these abnormalities interact has yet to be elucidated. Over the past decades, zebrafish have become an important model organism, especially in biomedical research. Several zebrafish models have been developed to recapitulate the characteristics of specific myeloid malignancies that provide novel insight into the pathogenesis of these diseases and allow the evaluation of novel small molecule drugs. This report will focus on illustrative examples of applications of zebrafish models, including transgenesis, zebrafish xenograft models, and cell transplantation approaches, to the study of human myeloid malignancies.

## 1. Introduction

Myeloid malignancies, including myeloproliferative neoplasms (MPNs), myelodysplastic syndrome (MDS), and acute myeloid leukemia (AML), are heterogeneous disorders characterized by uncontrolled proliferation or/and blockage of differentiation of abnormal myeloid progenitor cells [1]. MPNs are clonal hematopoietic stem cell disorders characterized by proliferation of one or more of the myeloid lineages. MDS is also a clonal hematopoietic disorder characterized by the simultaneous proliferation and apoptosis of hematopoietic cells, which leads to a normal state or hypercellularity of the bone marrow (BM) and pancytopenia in the peripheral blood (PB).

A substantial number of studies have revealed that MPN or MDS can evolve into AML in some patients [2]. AML is a heterogeneous disease that results from the clonal expansion of myeloblasts in the BM and PB and may involve only one or all myeloid cell lineages [3, 4]. Numerous recurrent gene fusions (such as t(15;17)/*PML-RARA*, t(8:21)/*AML1-ETO*, inv(16)/*CBFB-MYH11*, and t(9;11)/*MLL-MLLT3*) and point mutations (such as* NPM1*,* FLT3*,* KIT*, and* CEBPA*), associated with class I (proliferation advantage) and class II (differentiation blockage) mutations, have been identified over the past several decades. Recent comprehensive studies with whole-genome sequencing or whole-exome sequencing, RNA and microRNA sequencing, and DNA methylation analysis have successfully classified the related genes into nine categories based on the function of the mutated genes, including transcription factor gene fusions,* NPM1*, tumor suppressor genes, genes with chromosome modifiers, genes with DNA methylation, activated signaling genes, myeloid transcription factor genes, cohesion-complex genes, and spliceosome-complex genes [5]. However, the mechanisms which by these identified alterations interact to induce AML and distinguish driver from passenger mutations in leukemogenesis have yet to be elucidated.

The zebrafish is a popular research model in biomedical research fields, including embryonic development, human diseases, cancer studies, toxicity, and chemical screening [6]. From a genetic point of view, the current zebrafish genome has been fully sequenced, and many genes are conserved between the human and zebrafish genomes. The zebrafish genome is composed of 25 chromosomes and essentially contains the full vertebrate repertoire of genes. More importantly, approximately 84% of the human genes that cause diseases have a zebrafish ortholog [7]. Although zebrafish and mammalian hematopoietic organs belong to different sites, the genetic and cellular levels of hematopoiesis are conserved between these groups. Zebrafish carry their hematopoietic stem cells in the kidney marrow and have blood cell types similar to human beings [8, 9].

Compared to the mouse model, the zebrafish model is very suitable for large-scale genetic and high-throughput screening in many ways, and it allows more powerful induction of tumors by carcinogens [10]. The first zebrafish model of hematological malignancy was generated in 2003 using the mouse* Myc* gene driven by* recombinase activating gene 2* (*rag2*) promoters. This zebrafish model faithfully developed T-cell lymphoblastic leukaemia (T-ALL) that closely parallels the human disorder subtype [11]. Recently, the zebrafish system has been used to study genetic pathways and understand the pathogenesis involved in human cancers [12–14]. Thus, the zebrafish provides a unique model system to study disease mechanisms* in vivo*. This paper summarizes the benefits of using the zebrafish model to study myeloid leukaemogenesis, reviews current zebrafish models of specific myeloid malignancies, and gives future directions for zebrafish models in the study of human cancer. In addition, the bridge between basic science and translational research will be discussed.

## 2. Overview of Zebrafish Hematopoiesis

Hematopoiesis is a complex process that utilizes many transcription factors to form all of the blood cell lineages from common multipotent hematopoietic stem cells (HSCs). Various experimental tools and methods have been established to facilitate the understanding of hematopoiesis and blood-related disease mechanisms. The zebrafish is an ideal animal model to study hematopoietic development due to its experimental advantages. Although they possess some characteristics that are different from other vertebrates and mammals, such as the site of hematopoiesis, the lineages of blood cells as well as the transcriptional regulators associated with the fate of blood cells have been evolutionarily conserved [15, 16]. Similar to other vertebrates, two major waves of hematopoiesis, the primitive and definitive waves, sequentially occur in zebrafish hematopoiesis [17]. The first wave of definitive hematopoiesis produces a transient population of cells, termed erythroid myeloid progenitors (EMPs), in the posterior blood island (PBI). The population of primitive myeloid cells is predominantly different from that of hemangioblasts at the anterior lateral mesoderm (ALM) between 12 and 24 hours after fertilization (hpf) [18, 19]. However, the posterior lateral mesoderm (PLM) is the major location of primitive erythroid progenitors and some myeloid cells [20–23]. Beginning at 24 hpf, these primitive blood cells enter the circulation and are distributed throughout the embryo. The definitive wave of hematopoiesis begins at approximately 26–30 hpf, and multipotent HSCs emerge from the hemogenic endothelium that resides in the ventral aspect of the dorsal aorta in the aorta-gonad-mesonephros (AGM) region [21, 24, 25]. They then migrate to the posterior region of the tail-caudal hematopoietic tissue (CHT), along with the circulation, after 36 hpf, and differentiate into cells during that period of time [26, 27]. Ultimately, HSCs from the AGM and CHT seed the kidney marrow approximately 4 days after fertilization (dpf) and give rise to all lineages of blood cells for the remainder of adult life. The CHT is analogous to the mammalian fetal liver or placenta, while the kidney marrow is functionally equivalent to mammalian bone marrow. In addition, some of HSCs also seed the thymus, where lymphopoiesis is initiated at approximately 3 dpf, and remain permanently after the maturation of lymphocytes.

Several zebrafish transcription factors that regulate hematopoiesis have been identified. The early stage markers* gata2*,* lmo2*,* fli1*, and* scl* (*stem cell leukemia*) are master regulators that are coexpressed in both the ALM and PLM, where hemangioblast development occurs, from the 2nd to the 3rd somite stages [20, 21, 23]. These genes are expressed in the PLM and later in the intermediate cell mass (ICM) [28–31].* lmo2*,* fli1*, and* scl* may function as crucial factors necessary for normal erythroid and myeloid development [30–32]. In addition,* gata1* and* spi1* (*pu.1)* are also involved in primitive hematopoiesis. Gata1, a transcription factor essential for erythropoiesis, is first detectable in cells of the PLM at 12 hpf and then in the anterior ICM [33, 34]. Conversely, expression of* spi1*, an ETS transcription factor required for myeloid cell development, appears in the ALM and ICM between 16 and 30 hpf [35]. Later, these* spi1* expressing progenitor cells differentiate into macrophages and granulocytes that express* l-plastin* (*lcp1*) and myeloperoxidase (mpo), respectively [18, 36]. Runx1 has been shown to be required for definitive hematopoiesis based on knockdown experiments that resulted in reduced c-myb expression and lymphopoiesis, but it produces no apparent effect on the development of primitive hematopoiesis [24, 37–39].

## 3. Transgenic Technology for Studying Myeloid Malignancies Using Zebrafish

The available zebrafish transgenic technology has improved over the last two decades [40, 41]. Different systems have been used in transgenic zebrafish models, such as injection of linear DNA [40] or supercoiled plasmid DNA [41, 42] or injection of recombinant bacterial artificial chromosomes into embryos [42]. In recent years, a new transgenic technology related to* Tol2*-mediated transgenesis has been established. The* Tol2* element is a naturally occurring active transposable element found in vertebrate genomes. The* Tol2* transposon system is considered a useful gene transfer vector in organisms ranging from fish to mammals [43].* Tol2*-mediated transgenesis is an excellent method for creating transgenic zebrafish using coinjection of* Tol2* RNA, and the DNA fragment surrounded by the* Tol2* element transposon can be efficiently excised and integrated into the zebrafish genome [44, 45].

Constitutive expression systems have proven useful, but it is often observed that the expression of oncogenes may cause serious tumors and early lethality, preventing the complete characterization of their effects. Therefore, the use of an induction system can help to solve this problem. For example, Tet-On, Tet-Off, Mifepristone, Cre-loxP, Heat-shock, and GAL4-UAS inducible systems can be used. The duration and dosage of oncogene expression can be monitored with such a system, thus allowing the spatiotemporal control of gene expression [46].

**Figure 1:**
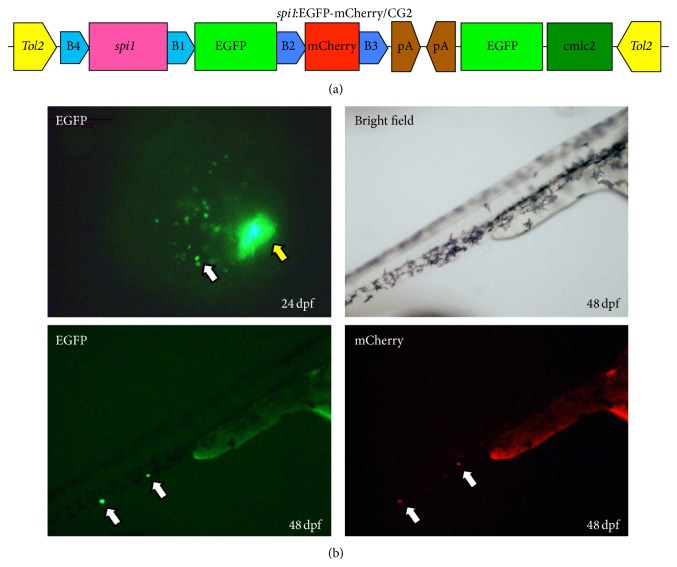
Expression of the* spi1*:EGFP-mCherry/CG2 transgenic fish. (a) Diagram of the* spi1*:EGFP-mCherry/CG2 construct that contains* Tol2* sequences and the cmlc2:GFP expression cassette. (b) Fluorescent images of TG (*spi1*:EGFP-mCherry/CG2) embryos at 24 or 48 hpf. The white arrowhead indicates* spi1* expressing myeloid cells and the yellow arrowhead fluorescence in heart.

A green fluorescent protein (GFP) reporter can be a useful marker for determining if mammalian promoters and ubiquitous or endogenous tissue-specific promoters can drive downstream transgene expressions in zebrafish [43]. It has been noted that* spi1* plays an important role in the development of myeloid (granulocytes and monocytes/macrophages) cells in zebrafish, and the* spi1* promoter can drive myeloid-specific expression in zebrafish. Ward et al. [47] and Hsu et al. [48] identified a 5.3 kb and a 9.0 kb promoter fragment of zebrafish sequences upstream of the* spi1* coding sequence that is sufficient to drive the expression of downstream genes. Recently, a transgenic fish line with a* spi1*:EGFP-mCherry/CG2 construct was established for studying the cooperation of various genetic aberrations found in myeloid malignancies (Figure 1), and this model may become a useful zebrafish model for exploring leukemogenesis in myeloid cells.

## 4. Myeloid Malignancies Animal Models in Zebrafish

*RAS* mutations associated with cancer frequently occur in patients with AML, suggesting a functional role for Ras in leukemogenesis.* AML1/ETO* rearrangements are detected frequently in AML, especially M2, and are associated with a relatively good prognosis [49].* K-RASG12D* interacts with* AML1/ETO* to induce acute monoblastic leukemia in a mouse model [50]. The* NUP98-HOXA9* fusion oncogene is related to an inferior prognosis in de novo and treatment-related AML and induces AML in mouse models [51].* NUP98-HOXA9* is associated with increased cell proliferation and survival as well as drug metabolism [52]. The* MOZ/TIF2* fusion oncogene was described in a specific subgroup of AML that represents approximately 5% of M4/M5 AML [53]. The transforming properties of* MOZ/TIF2* have been demonstrated in mouse committed myeloid progenitors* in vitro* and* in vivo* [54, 55]. Constitutive activation of* Stat5* has been observed in hematological malignancies and is often triggered by leukemic oncoproteins, such as* Tel-Jak2* and* Bcr-Abl*, and* Stat5* has been shown to be involved in mediating the leukemic effects [56, 57]. Amplification of* MYCN (N-Myc)* is frequently found in AML patients and is considered a well-established poor prognostic marker in this disease. Overexpression of* MYCN* rapidly causes AML in mouse models [58, 59]. In AML patients,* FLT3* and* NPM* have important prognostic implications on the treatment outcome. Patients with mutated* NPM* have a favorable outcome, while patients with mutations in the* FLT3* gene generally have a poor prognosis.* NPM1* and* FLT3-ITD* mutations interact to induce AML in mouse models [60].

Several zebrafish models associated with myeloid malignancies have been reported. In a transgenic zebrafish model,* K-RAS*G12D was expressed under the actin promoter and induced MPNs [61]. Induction of* NUP98-HOXA9* fusion genes in transgenic zebrafish under the* spi1* promoter leads to MPNs at 19–23 months of age. However, in contrast to the mouse models, none of the* NUP98-HOXA9* transgenic fish developed AML [62–64]. Lewis et al. addressed the transient expression of constitutively active* Stat5* (*H298R/N714F*-mutant) in zebrafish, which leads to increased numbers of early and late myeloid cells, erythrocytes, and B cells [65]. Bolli et al. found that transient expression of the* NPM1 *mutant in zebrafish increases the number of definitive hematopoietic cells, including erythromyeloid progenitors, in the posterior blood island and* c-myb*/cd41+ cells in the ventral wall of the aorta [66].

In a transgenic zebrafish model, the* tel-jak2a* fusion oncogene was expressed in embryos under the control of the* spi1* promoter, which resulted in disruption of embryonic hematopoiesis, including anemia and expansion of the myeloid compartment [67]. Transient expression of* AML1-ETO* under the control of a CMV promoter in zebrafish embryos caused disruption of normal hematopoiesis, aberrant circulation, internal hemorrhages, and cellular dysplasia [24]. Induction of* AML1-ETO* transgenic zebrafish with the* hsp70* promoter led to reprogramming of the multipotent hematopoietic progenitor cells from the erythroid cells to the myeloid cells in primitive hematopoiesis and disruption of definitive hematopoiesis in embryonic zebrafish [68].

Transient expression of* FLT3-ITD* in zebrafish embryos induced ectopic myeloid cell expansion and clustering, which were ameliorated by AC220 and associated with* stat5*,* erk1/2*, and* akt* phosphorylation. Overexpression of the* FLT3-ITD/TKD* (D835Y) double mutation in zebrafish embryos conferred resistance to AC220 treatment. This zebrafish model may be useful for assessing the pathogenic significance and therapeutic potential of novel gene mutations [69].

In a transgenic zebrafish model of AML,* MYCN*, under the control of a* CMV* minimal promoter and a* MOZ/TIF2* fusion gene generated by the inv(8)(p11q13) chromosomal abnormality expressed under the* spi1* promoter, resulted in altered hematopoiesis and was characterized by invasion of the kidney marrow by immature myeloid cells [58, 70]. Until now, only two published zebrafish AML models have developed overt leukemia (Table 1).

Recently, a transgenic zebrafish model that expresses* AML1-ETO* oncogenes has been reported as an excellent animal model for uncovering new therapeutic targets involved in oncogene-regulated hematopoietic differentiation [71]. According to these studies, the use of human genes produces greater fidelity and reliability in the translation of results associated with the interaction of molecular signaling pathways and disease therapy. In conclusion, the zebrafish is an excellent animal model for elucidating the mechanisms of leukemogenesis and provides an effective drug screening platform.

## 5. Drug Screening in Zebrafish Using Xenograft Model

One of the most extensively used* in vivo* animal models in the investigation of molecular mechanisms is xenotransplantation in immune-deficient animals. The zebrafish genome is nearly 100% homologous to the human genome in key domains [72]. The xenograft animal model has been utilized to investigate tumor biology, especially tumor cell proliferation, invasion, metastasis, and angiogenesis for decades, and the zebrafish model has been used as an alternative to mammalian models to assess the efficacy and toxicity of cancer drugs since 2005 [73].

Engrafting of human tumor cells into the yolk sac of zebrafish embryos at 48 hpf can be used as a xenograft model of zebrafish due to the lack of an adaptive immune response at this stage [74]. To achieve maximum embryo transparency, embryos were incubated in egg medium with 0.3% phenylthiourea (PTU) to prevent pigment formation. As in mouse systems, the* in vivo* spatial resolution of the adult mouse is limited due to the normal opacification of the skin and subdermal structures. However, the body of zebrafish embryos is transparent, which allows the observation of labeled tumor cells and evaluation of the effects of cancer drugs. Due to the size of zebrafish embryos, high-throughput drug screening can be conducted in a 96-well format [74]. Several reports have verified that the results from zebrafish xenograft models are similar to those from mouse models [75, 76]. Taken together, the findings suggest that the zebrafish model may be a rapid, simple, sensitive, and reproducible xenograft model compared to the mouse model (Table 2).

Leukemia cells labeled with CM-Dil, a lipophilic fluorescent tracking dye, were injected into 2-day-old zebrafish larvae and used to evaluate the efficacy of imatinib and all-trans retinoic acid (ATRA) on the proliferation of K562 cells and NB4 cells, respectively [6], and these cells have also been used in* ex vivo* cell proliferation assays [77]. A novel phenotype-based* in vivo* screening method that uses leukemia stem cells (LSCs) xenotransplanted into zebrafish has been demonstrated. Aldehyde dehydrogenase-positive (ALDH+) cells reflecting LSCs were implanted into young zebrafish at 48 hpf, and the efficacy of various therapeutic agents was evaluated by high-content imaging [78]. From the transplanted tumor clusters, the number of migrating tumors and their areas were measured within concentric rings at a defined distance from the main tumor by calculating the area, total luminance value, and average radius of the tumor [62]. For cell proliferation assays, positive embryos were divided into two groups; one group was maintained at 35°C for 24 h, while the other group was incubated with or without drug for 72 h. At the end of each time period, the embryos were enzymatically dissociated into a single cell suspension, and the number of fluorescent cells in the suspension was counted. The number of fluorescent cells present at 72 h divided by the number of fluorescent cells present at 24 h represents the fold increase in the cell number [78]. These studies established the efficacy of a zebrafish xenograft platform as a rapid assessment of the effect of novel compounds on leukemia cell proliferation* in vivo*. Taken together, these findings suggest that the zebrafish xenograft model can be used as a platform for drug screening, a tool to rapidly assess the efficacy of novel compounds on the proliferation of human leukemia cells* ex vivo* and for providing information for subsequent preclinical mouse studies and clinical trials.

## 6. Zebrafish Models for Hematopoietic Cell Transplantation Biology

Hematopoietic stem cells are the source of all the blood cells needed by an organism during its lifetime. The study of hematopoiesis has been markedly facilitated by hematopoietic stem cell transplantation (HSCT), which involves transplanting donor blood cell populations into recipient animals. It is known that immune-matching is not required in embryonic recipients less than 5 days after fertilization because thymic development does not occur until that time in zebrafish [79]. Therefore, HSCT could be applied in experiments related to the development of quantitative long-term repopulating assays and the generation of histocompatible zebrafish lines [80].

The first hematopoietic cell transplantation in zebrafish revealed that hematopoietic cell transplantation could rescue multilineage hematopoiesis in embryonic lethal* gata1* ^−/−^ mutants over six months of age [15]. The 2-day-old embryos were used as transplant recipients to partly circumvent graft rejection by performing short-term multilineage hematopoietic engraftment [15]. Subsequently, Traver et al. demonstrated an allogeneic HSCT into adult recipient zebrafish conditioned by a sublethal dose of gamma irradiation and established transplantation assays to evaluate the function of HSCs in zebrafish [81]. Self-renewal is a feature of cancer, and Smith et al. initially demonstrated high-throughput imaging methods to experimentally assess cell transplantation and evaluate the gene pathways involved in cancer self-renewal [82]. However, these approaches require donor cells from the same strain of syngeneic zebrafish or the recipient immune system to be transiently ablated by whole-body *γ*-irradiation before transplantation [15, 81, 82], making long-term engraftment studies difficult. In 2011, lethally irradiated animals could be rescued by transplantation of whole kidney marrow cells. The key zebrafish genes at the major histocompatibility complex locus on chromosome 19 were identified, and transplantation of hematopoietic stem cells was successfully performed based on immune-matching [80]. Recently, *rag*2^E450fs^ mutant zebrafish, which have reduced numbers of functional T and B cells, were successfully created, and these animals could be used as universal recipients for allograft cell transplantation. This is the first established immunocompromised zebrafish model, and it may produce a new era of stem cell self-renewal and large-scale cell transplantation studies [83]. These advances provide unique opportunities to investigate the mechanisms of engraftment.

## 7. Concluding Remarks

AML is a heterogeneous disorder characterized by acquired genetic changes in hematopoietic progenitor cells. Numerous recurrent gene fusions and point mutations have been identified over the past several decades. However, the mechanism by which these identified alterations interact to induce AML and distinguish driver from passenger mutations in leukemogenesis remains unknown. Chemotherapy is currently used as the standard treatment for AML, except for AML-M3, which shows a good therapeutic response when treated with all-trans retinoic acid (ATRA) or arsenic trioxide, and the majority of AML patients relapse after complete remission or acquire drug resistance, indicating the need for efficacious therapeutic strategies. Several small molecule inhibitors have been developed that showed efficacy in preclinical studies; however, approval of these agents for clinical treatment is still challenging.

The zebrafish genome has been fully sequenced and has a substantial number of conserved genes compared to the human genome. The genetic and cellular levels of hematopoiesis are conserved between these organisms. Several zebrafish models associated with myeloid malignancies have been previously reported, including transgenic zebrafish that exhibit* K-RASG12D*,* NUP98-HOXA9*,* Stat5 (H298R)*,* Stat5 (N714F)*,* NPM1*,* tel-jak2a*,* AML1-ETO*,* FLt3-ITD*,* FLT3-TKD* (D835Y),* MYCN*, or* MOZ/TIF2 *expression. Several zebrafish transgenic technologies have also been introduced, including* Tol2*-mediated transgenesis and GFP-mCherry transgenic lines driven by a myeloid-specific* spi1* promoter. In addition, xenograft zebrafish models used for high-throughput drug screening and leukemia xenotransplantation in zebrafish used for* in vivo* chemotherapy response assays have also been addressed.

## Figures and Tables

**Table 1:** Zebrafish animal models of myeloid malignancies.

Gene name	Promoter and expression construct	Type	Refs
*MYCN *	MYCN-HSE-EGFP	AML	[58]

*MYST3/NCOA2 *	spi-1-MYST3/NCOA2-EGFP	AML	[70]

*kRASG12D *	B-actin-LoxP-EGFP-LoxP-kRASG12D; hsp70-Cre	MPD	[61]

*NUP98-HOXA9 *	spi-1-loxP-EGFP-loxP-NUP98-HOXA9; hsp70-Cre	MPN	[62]

*Stat5.1 *mutants	Constitutively active mutants of Stat5.1	Tumor-like lesions; increased numbers of early and late myeloid cells, erythrocytes, and B cells	[65]

*NPM1* mutant	pCS2cmv-NPM1c-EGFP	No AML; increased numbers of definitive hematopoietic cells	[66]

*tel-jak2a *	pCS2cmv-Flag-tel-jak2a; spi-1-Flag-tel-jak2a	No AML; anemic; perturbed intermediate cell mass; accumulation of large cells near the heart	[67]

*AML1 *	pCS2cmv-runx1	No AML; enlarged heart and ectopic blood	[24]

*AML1-ETO *	pCS2cmv-RUNX1-CBF2T1	No AML; defective development of blood and circulation and internal hemorrhaging	[24]

*AML1-ETO *	hsp-AML1-ETO	No AML; loss of gata1 hematopoietic cells in the posterior blood islands	[68]

*AML1-ETO* + Gro3 MO	hsp-AML1-ETO	No apparent AML; enhanced the accumulation of blast cells	[68]

*FLT3-ITD *	CMV-FLT3-ITD-T2a-EGFP	Ectopic myeloid cell expansion	
	CMV-FLT3-TKD-T2a-EGFP	Ectopic myeloid cell expansion resistant to AC220	[69]
	CMV-FLT3-ITD-TKD-T2a-EGFP	Conferred resistance to AC220 treatment	

AML: acute myeloid leukemia; MPD: myeloproliferative disorder; MPN: myeloproliferative neoplasm; MO: morpholino.

**Table 2:** The advantages and weaknesses in the xenograft model of zebrafish and mouse.

	Item	Zebrafish	Mouse
Strengths	Maintenance cost	Low	Available
	Maintenance space	Small	Large
	Offspring	Large number	Less number
	Immune system	Lack in early zebrafish embryos	Innate and adaption
	Observation	Visualization (transparency and transgenic lines)	Limitation
	Readout time	Hours to days	Days to months
	Cell numbers required for xenotransplantation per animal	Less	Large
	High-throughput drug screening	Available	Limitation

Weaknesses	Size of organs/vessels	Small	Large
	Body temperature	Low	High
	Lack of organs	Breast, lung, etc.	
	Zebrafish antibodies	Limitation	More
	Adult immune-permissive lines	Unavailable	Available
